# Optic nerve sheath calcification in a patient with chronic kidney disease: an unusual orbital manifestation

**DOI:** 10.1590/2175-8239-JBN-2025-0085en

**Published:** 2025-06-30

**Authors:** Lara Hemerly De Mori, Nina Ventura, Diogo Goulart Corrêa

**Affiliations:** 1 Universidade do Estado do Rio de Janeiro, Hospital Universitário Pedro Ernesto, Departamento de Diagnóstico por Imagem, Rio de Janeiro, RJ, Brasil.; 2 Universidade Federal do Rio de Janeiro, Hospital Universitário Clementino Fraga Filho, Departamento de Radiologia, Rio de Janeiro, RJ, Brasil.; 3 DASA, Clínica de Diagnóstico por Imagem (CDPI), Departamento de Radiologia, Rio de Janeiro, RJ, Brasil.

A 66-year-old man with chronic kidney disease (CKD) of hypertensive origin on hemodialysis for three years presented with bilateral amaurosis for two months. Fundoscopy was normal. Brain computed tomography revealed bilateral calcifications in the optic nerve sheath (Figure 1).

**Figure 1:**
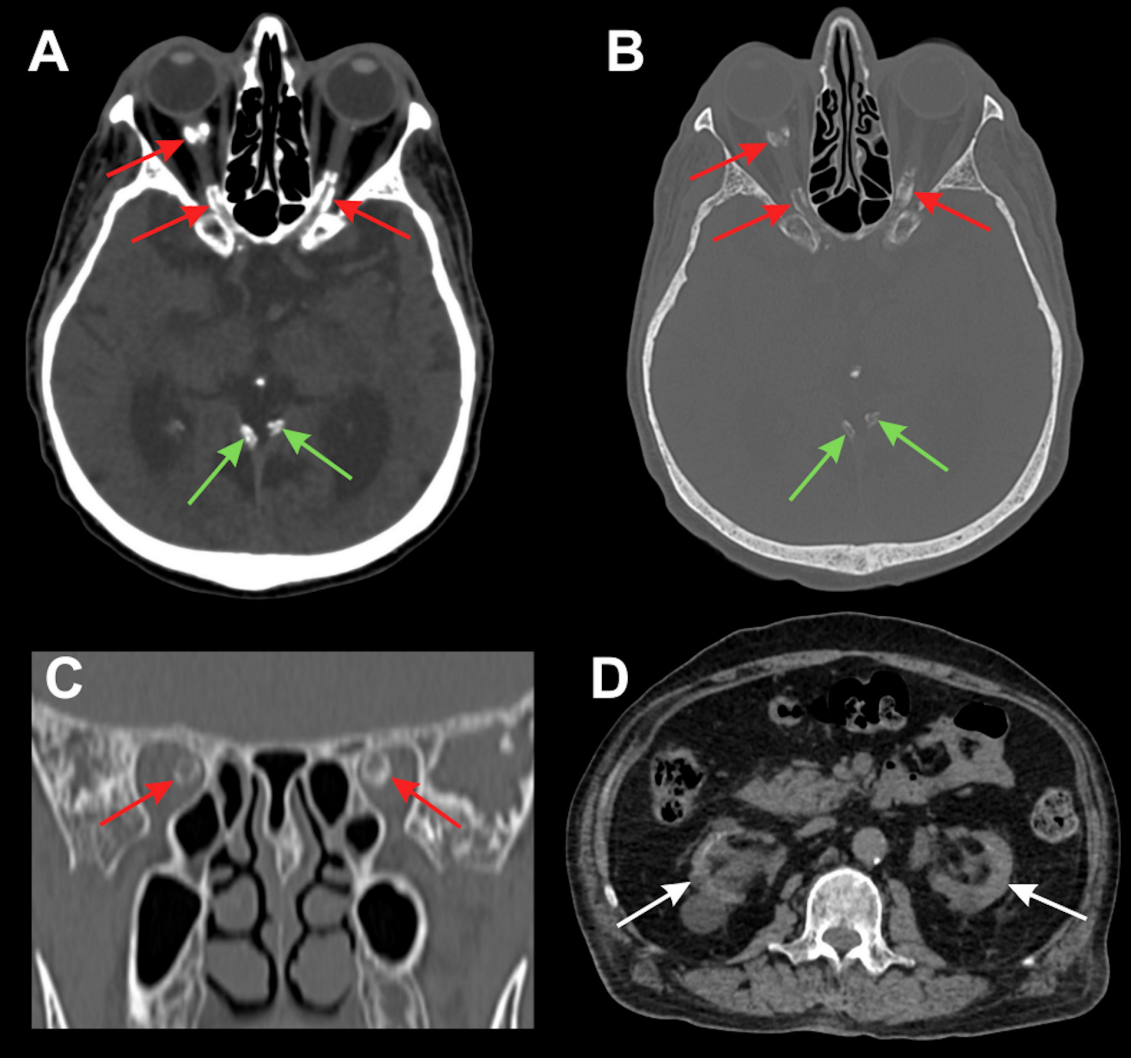
Optic nerve sheath calcifications due to hyperparathyroidism secondary to chronic kidney disease. Brain computed tomography (CT) revealed bilateral calcific foci in the optic nerve sheaths (arrows in A, B, and C), in the brain parenchyma window (A), and in the bone window (B and C). Meningeal calcifications were also noted in the tentorium cerebelli (green arrows in A and B). Imaging evaluation of the kidneys on upper abdominal CT showed a marked reduction in renal parenchymal thickness and multiple bilateral cortical cysts (arrows in D), in addition to some aortic calcifications.

Disorders of phosphorus and calcium metabolism due to hyperparathyroidism are common in CKD and can cause metastatic calcium deposition in soft tissues^1,2^. In this case, laboratory tests revealed a parathyroid hormone (PTH) level of 105 pg/mL, serum calcium of 12.5 mg/dL, serum phosphate of 3.8 mg/dL, alkaline phosphatase (ALP) of 192 U/L, and 25-hydroxyvitamin D of 17 ng/mL, suggesting a rare orbital manifestation of CKD-related mineral and bone manifestation. Meningioma, glioma, *phthisis bulbi*, and previous hemorrhages are some of the main differential diagnoses^3,4^.

## Data Availability

Available upon relevant requests.
