# Supplementary material for: Optic nerve sheath calcification in a patient with chronic kidney disease: an unusual orbital manifestation
Source: J Bras Nefrol. 2025 Jun 30;47(3):e20250085. doi: 10.1590/2175-8239-JBN-2025-0085en (PMC12208610; doi:10.1590/2175-8239-JBN-2025-0085en)
Supplement: Translation

# Translation: Calcificação da bainha do nervo óptico em um paciente com doença renal crônica: uma manifestação orbitária incomum

**DOI:** 10.1590/2175-8239-JBN-2025-0085pt

**Authors:** Lara Hemerly De Mori, Nina Ventura, Diogo Goulart Corrêa

**Affiliations:** 1 Universidade do Estado do Rio de Janeiro, Hospital Universitário Pedro Ernesto, Departamento de Diagnóstico por Imagem, Rio de Janeiro, RJ, Brasil.; 2 Universidade Federal do Rio de Janeiro, Hospital Universitário Clementino Fraga Filho, Departamento de Radiologia, Rio de Janeiro, RJ, Brasil.; 3 DASA, Clínica de Diagnóstico por Imagem (CDPI), Departamento de Radiologia, Rio de Janeiro, RJ, Brasil.

Homem, 66 anos, com doença renal crônica (DRC) de etiologia hipertensiva, em hemodiálise há três anos, evoluiu com amaurose bilateral há dois meses. A fundoscopia encontrava-se normal. Foi realizada tomografia computadorizada do crânio, que revelou calcificações na bainha do nervo óptico bilateralmente (Figura 1).

**Figura 1:** Calcificações da bainha dos nervos ópticos por hiperparatireoidismo secundário à doença renal crônica. Tomografia computadorizada (TC) do crânio revelou focos cálcicos na bainha dos nervos ópticos bilateralmente (setas vermelhas em A, B e C), tanto na janela para o parênquima cerebral (A), quanto na janela óssea (B e C). Calcificações meníngeas também foram observadas no tentório cerebelar (setas verdes em A e B). A avaliação tomográfica dos rins na TC do abdome superior mostrou redução importante da espessura do parênquima renal e múltiplos cistos corticais bilateralmente (setas em D), além de algumas calcificações aórticas.

Distúrbios do metabolismo do fósforo e do cálcio, decorrentes do hiperparatireoidismo, são comuns na DRC e podem causar deposição metastática de cálcio em tecidos moles^1,2^. Neste caso, os exames laboratoriais revelaram níveis de paratormônio (PTH) de 105 pg/mL, cálcio sérico de 12,5 mg/dL, fosfato sérico de 3,8 mg/dL, fosfatase alcalina (FA) de 192 U/L e 25-hidroxivitamina D de 17 ng/mL, sugerindo uma manifestação orbitária rara do distúrbio mineral e ósseo relacionado à DRC. Meningioma, glioma, *phthisis bulbi* e hemorragias prévias compõem alguns dos principais diagnósticos diferenciais^3,4^.

## Data Availability

Disponíveis mediante solicitações pertinentes.
